# Bridging Literature and Real-World Evidence: External Evaluation and Development of Fluoxetine Population Pharmacokinetics Model

**DOI:** 10.3390/pharmaceutics17121516

**Published:** 2025-11-24

**Authors:** Bing Han, Nuo Xu, Chen Ma, Gehang Ju, Xie Xi, Cheng Qian, Nan Guo, Xin Liu, Xiao Zhu, Cong Li, Li Liu

**Affiliations:** 1 School of Pharmaceutical Sciences, Fudan University, Shanghai 201203, China; hbshcn@fudan.edu.cn (B.H.); nuoxu22@m.fudan.edu.cn (N.X.); machen@fudan.edu.cn (C.M.); 25211030098@m.fudan.edu.cn (C.Q.); liux24@m.fudan.edu.cn (X.L.); xiaozhu@fudan.edu.cn (X.Z.); 2 Department of Clinical Pharmacology and Clinical Trial, The Second People’s Hospital of Hunan Province (Brain Hospital of Hunan Province), Changsha 410007, China; 3 Institute of Big Data, Central South University, Changsha 410083, China; jugehang1995@163.com; 4 Department of Medical Laboratory, Hunan Prevention and Treatment Institute for Occupational Diseases, Affiliated Prevention and Treatment Institute for Occupational Diseases of University of South China, Changsha 410007, China; xiexichn@163.com; 5 Minhang Hospital, Fudan University, Shanghai 201100, China; guon@fudan.edu.cn; 6 State Key Laboratory of Advanced Drug Formulations for Overcoming Delivery Barriers, Fudan University, Shanghai 201203, China

**Keywords:** fluoxetine, model-informed precision dosing, population pharmacokinetics, external evaluation

## Abstract

**Background:** Fluoxetine is widely prescribed to treat depression but exhibits high inter-individual and inter-ethnic pharmacokinetic (PK) variability. Most published population pharmacokinetic (PopPK) models were derived from Western patients, and their applicability to Chinese patients remains uncertain. **Methods:** A systematic review of the published fluoxetine PopPK models was carried, and the relevant demographic and model parameters were extracted. A retrospective real-world dataset from Chinese psychiatric patients was then collected. External evaluation was conducted to assess the model’s predictive performance. Subsequently, a joint parent–metabolite PopPK model was developed to better characterize fluoxetine and its active metabolite norfluoxetine in Chinese patients. Finally, Monte Carlo simulations were performed to evaluate once-daily dosing regimens of 10–60 mg for 30 days, focusing on the probability of achieving target (PTA) steady-state trough concentrations (C_min,ss_). **Results:** Two published PopPK models were identified and externally evaluated using data from 198 Chinese patients with 241 fluoxetine and 241 norfluoxetine plasma concentrations. Both models were shown to have prediction discrepancy. The parent drug–metabolite model was used to describe the characteristics of fluoxetine and norfluoxetine in the Chinese population. Sex was identified as the significant covariate, and males exhibited a 16.5% higher clearance than females. The simulation results indicate that the maximum effective dose for females is 30 mg once daily, and for males, it is 40 mg once daily. **Conclusions:** This study provides the first comprehensive external evaluation of published fluoxetine PopPK models and establishes a tailored joint model that incorporates sex effects to explain trough variability in Chinese psychiatric patients. The findings support 30–40 mg once daily as a practical dosing range for Chinese adults and adolescents, with males more likely to require the higher dose.

## 1. Introduction

Depression is a complex psychiatric disorder characterized by a constellation of symptoms, including depressed mood, cognitive impairment, and so on. Adolescence represents a particularly vulnerable developmental stage for the onset of depression. Epidemiological evidence suggests that the risk of developing depression among adolescents aged 10–19 years worldwide can be as high as 34% [1,2]. Fluoxetine is considered the first-line pharmacological treatment for major depressive disorder in adults and adolescents [3]. However, one study reported that the remission rate of the initial treatment was only 25–30%, and the cumulative remission rate after multiple treatment steps was approximately 40–50%, highlighting the necessity of individualized dosing for adolescent patients with depression [4,5,6].

Therapeutic drug monitoring (TDM) is an established approach for optimizing and assessing drug therapy; however, its target ranges vary across populations. In adults, TDM typically focuses on the active moiety trough concentration (fluoxetine + norfluoxetine, C_min,ss_) within 120–500 ng/mL, whereas pediatric and adolescent data suggest narrower preliminary ranges of approximately 208–328 ng/mL [7,8]. Notably, real-world cohorts report that almost 40% of patients fall outside recommended windows after standard dosing, consistent with suboptimal target attainment [9]. This may be largely attributable to the considerable pharmacokinetic variability of fluoxetine. Fluoxetine’s pharmacokinetics complicate dose individualization, characterized by high oral bioavailability, extensive protein binding, large distribution volume, and long elimination half-lives (4–6 days) [10].

Inter-ethnic differences further exacerbate the challenges of precision dosing. East Asian populations, including Chinese, have higher frequencies of *CYP2C19* loss-of-function alleles (*2, *3) and distinct *CYP2D6* variant/structural, variant patterns (e.g., *10 and *36-*10 tandems), which can markedly alter parent–metabolite ratios and overall exposure [11]. Controlled studies in Chinese volunteers demonstrated that *CYP2C19* poor metabolizers had a significantly higher fluoxetine maximum plasma concentration (C_max_) (46%) and area under curve (AUC) (128%) than extensive metabolizers after a single dose [12]. Pharmacogenetic implementation guidelines highlight these genotype effects for SSRIs and the implications for dose optimization [13]. Collectively, such cross-population differences raise concerns about the direct applicability of Western-derived models to Chinese patients [14,15,16].

Precision dosing guided by model repository represents a highly valuable tool for clinical practice. By leveraging model repository, fluoxetine’s pharmacokinetic characteristics could be systematically characterized, enabling cross-population comparisons of target attainment [17,18]. Model-informed precision dosing (MIPD) optimizes individualized dosing by integrating drug concentrations with multiple covariates that influence drug exposure. Although existing mechanistic models have demonstrated utility in elucidating pharmacokinetic variability and informing precision dosing, comprehensive integrative studies remain lacking [19,20,21].

There are already successful application cases of MIPD, including guiding missed or delayed dose administration through model repositories [22], optimizing sampling strategies in pediatric populations, and predicting individual drug exposure using maximum a posteriori Bayesian estimation [23]. Accordingly, to bridge the current knowledge gap, the present study aimed to (i) assemble a repository of published fluoxetine population pharmacokinetic (PopPK) models to identify limitations in the existing work; (ii) perform external evaluation using Chinese plasma PK data to assess cross-ethnic predictive performance; and, (iii) if substantial bias was observed, develop and optimize a joint fluoxetine–norfluoxetine model tailored to Chinese patients for simulation-based dosing guidance and clinical dose recommendations.

## 2. Methods

### 2.1. Search Strategy

A systematic literature search was performed in the PubMed, Web of Science, and Embase databases, with the literature screened from database inception to 7 May 2025. The search terms included MeSH terms plus Entry terms. Entry terms included Fluoxetin, Prozac, Lilly110140, Fluoxetine Hydrochloride, and Sarafem. BH and GHJ conducted the literature screening work back-to-back. The paper management software was EndNote (Version 21; Thomson Scientific, Box Hill, Victoria, Australia). The eligible PopPK article should meet the following inclusion criteria: (1) study population: human; (2) modelling approach: a parametric nonlinear mixed-effects model; (3) languages: published in English. We excluded the following: (1) type of paper: reviews or methodology articles (only describe the modeling strategy without reporting the model PK parameters); (2) duplicated work; (3) samples were breast milk; (4) non-parametric modeling or lack of key PK parameters. During the article screening phase, if the specific methods described in a study were clearly non-clinical, utilized models other than PBPK or PopPK, employed non-parametric methods, were published in a non-English language, or otherwise failed to provide content relevant to the investigational drug of this study, the study was classified as an irrelevant study.

### 2.2. Data Extraction and Quality Assessment

Two authors independently extracted the following information from eligible articles: (1) baseline data: demographic data (e.g., country, age, body weight, sex, etc.); (2) study characteristics: dosing regimens, sampling strategies, bioanalytical methods, the lower limit of quantification, etc.; (3) PopPK characteristics: model parameters, structural models, covariate screening methods, and investigated and included covariates.

The assessment of the quality of the final included studies was performed by utilizing a 33-item checklist consisting of 5 categories, according to a previous PopPK systematic review quality assessment method [23,24]. This checklist was designed to assess the essential components required for the reporting of clinical PK studies. For each item, one point was assigned if the involved literature met the criteria, whereas incomplete data were assigned 0.5 points. If the item did not meet the criteria, it was assigned 0 points. To evaluate the quality of each PopPK study, compliance was calculated using the following equation:

$$Compliance\left(\%\right)=\left(\frac{sum\;of\;items\;reported}{sum\;of\;all\;items}\right)*100\%$$

### 2.3. Dataset Preparation

Retrospective data were obtained from the medical records of patients treated with fluoxetine at Hunan Brain Hospital between 2021 and 2024. This study was approved by the Institutional Review Board (IRB) of the Second People’s Hospital of Hunan Province (Ethics Approval No. K2025042) and complied with the principles of the Declaration of Helsinki. Patients were eligible for inclusion if they had complete dosing histories, demographic data, and TDM records. All plasma samples were collected at steady state, prior to the next scheduled dose, and fluoxetine and norfluoxetine concentrations were measured using a validated LC–MS/MS method at Jiangsu Qlife Medical Technology Group Co., Ltd., Nanjing, China. The lower limit of quantification was 1 ng/mL. Importantly, none of the patients included in this external evaluation dataset had contributed data to the development of any of the previously published fluoxetine PopPK models under evaluation.

### 2.4. External Evaluation Process

To assess the external predictive performance and extrapolation capacity of each published model, model-based simulations were conducted using maximum a posteriori (MAP) Bayesian estimation. Since both published studies provided only the pharmacokinetic parameters for fluoxetine, the external evaluation in this work focused solely on assessing the extrapolation performance of the fluoxetine models. Although Panchaud et al. collected data for norfluoxetine, these data were excluded from their final model development due to the limited number of observations. Accordingly, norfluoxetine was not included in our external evaluation process. Recorded dosing regimens, sampling times, and covariates from the external dataset were used to compute both individual- and population-level predicted concentrations. When model-specified covariates were unavailable, mean or median values from the original studies were substituted. Visual and quantitative diagnostics were applied. Goodness-of-fit (GOF) plots were used to compare observed concentrations (C_obs_) with population-predicted (C_pred_) and individual-predicted (C_ipred_) values. Predictive accuracy was quantified using the following metrics: (1) prediction error (PE) for each observation (Equation (1)); (2) mean prediction error (MPE) (Equation (2)); (3) relative root mean squared error (RMSE) (Equation (3)). Additionally, normalized prediction distribution errors (NPDE) were computed using 1000 Monte Carlo simulations with the “npde” package (version 2.2). The NPDE values were assessed using three statistical tests: the Wilcoxon signed-rank test (H_0_: mean = 0), Fisher’s variance test (H_0_: variance = 1), and the Shapiro–Wilk test (H_0_: normal distribution).

(1) $$PE=\frac{C_{i,pred}-C_{obs}}{C_{obs}}$$

(2) $$MPE=\frac{1}{N}\sum_{i=1}^{N}PE$$

(3) $$RMSE=\sqrt{\frac{1}{N}\sum_{i=1}^{N}{PE}^{2}}$$

### 2.5. Development of Fluoxetine PopPK Model

PopPK analysis of fluoxetine and its active metabolite norfluoxetine was conducted using NONMEM^®^ 7.5 (ICON Development Solutions, San Antonio, TX, USA), with Perl-speaks-NONMEM (PsN, version 5.2.6; Uppsala University, Uppsala, Sweden) for model evaluation and data management. The model was developed using the same dataset as that used for the external evaluation, aiming to optimize model performance specifically for Chinese patients. The structural model for fluoxetine and norfluoxetine was developed following the standardized workflow [22]. Given the clinical data limitation (only steady-state trough concentrations were available), the absorption process could not be reliably estimated. Therefore, the absorption rate constant (ka) was fixed to literature-derived values (0.3 h^−1^) from the model repository to ensure model identifiability.

Covariate selection followed a stepwise approach, with forward inclusion (*p* < 0.01) and backward elimination (*p* < 0.001). Candidate covariates were derived from patients’ demographic data, including body weight, sex, and other clinically relevant parameters. Inter-individual variability (IIV) was modeled using exponential error structures, while residual unexplained variability (RUV) was described by a combined additive-proportional error model.

To ensure robust model performance and data fitting, internal validation was conducted using GOF plots and visual predictive checks (VPCs). Specifically, the stability of the final model was evaluated using a resampling method with replacement (bootstrap). In this procedure, the modeling dataset was repeatedly resampled with replacement under an appropriate stratified design to generate 1000 new datasets for model re-estimation. The distribution of parameter estimates obtained from these resampled datasets was then examined. If the parameter estimates from the original dataset were close to the median of the bootstrap estimates and fell within the 95% confidence interval of the bootstrap-derived parameter estimates, the model was considered stable. These procedures confirmed the model’s stability and appropriateness for the dataset. The final model code is provided in the Supplementary Materials.

### 2.6. Dose Optimization via Monte-Carlo Simulations

Monte Carlo simulations were performed to evaluate the impact of different dosing strategies on drug exposure in the target population. Simulations were conducted using the parameter estimates from the final joint parent–metabolite model for fluoxetine and norfluoxetine. Virtual patient populations (n = 1000 per scenario) were generated by resampling covariate distributions from the observed dataset to preserve the real-world variability. For each virtual patient, PK profiles were simulated under multiple dosing regimens, including standard and alternative strategies (e.g., different total daily doses). From these simulated profiles, C_min,ss_ was calculated at steady state (defined as ≥5 elimination half-lives). The proportion of individuals meeting pre-defined therapeutic exposure targets (fluoxetine plus norfluoxetine 120–500 ng/mL) was determined for each dosing regimen [8]. Comparisons across regimens were made to identify dosing strategies that maximized the proportion of patients achieving therapeutic exposure while minimizing the risk of excessive exposure. Simulation outcomes were summarized descriptively and visualized using probability–target attainment (PTA) plots based on R.

## 3. Results

### 3.1. Study Identification

**Figure 1:**
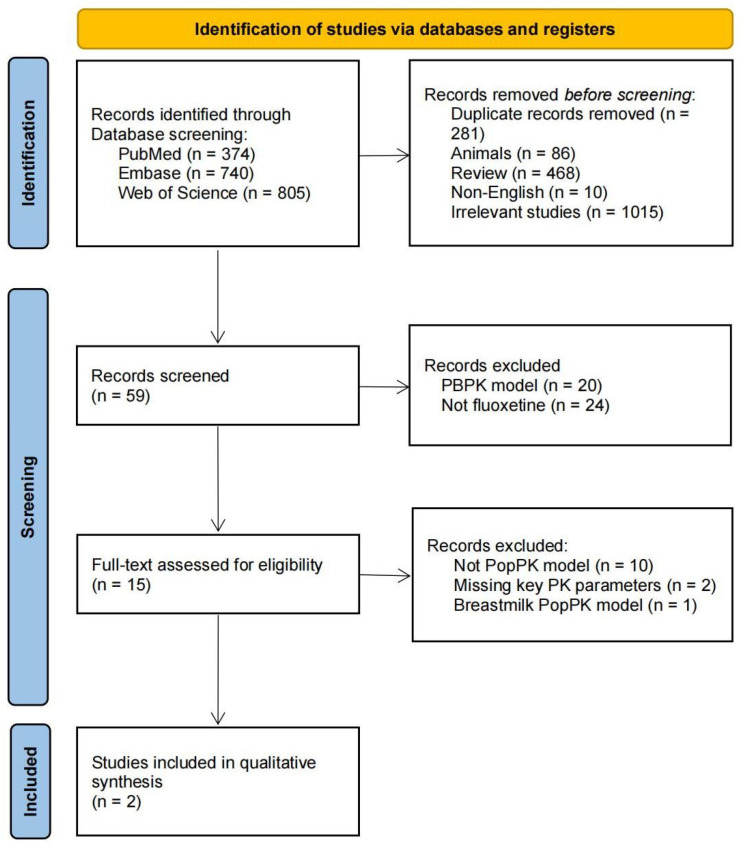
PRISMA flow diagram for the identification of fluoxetine PopPK studies. Notes: PRISMA Preferred Reporting Items for Systematic Reviews and Meta-analysis.

A total of 1919 articles were retrieved from the databases (PubMed: n = 374; Embase: n = 740; Web of Science: n = 805). After removing 281 duplicates, 1638 unique records remained. Initial screening excluded 86 animal studies, 468 review articles, 10 non-English reports, and 1015 studies on irrelevant topics, leaving 59 articles for detailed screening. After systematic screening and eligibility assessment, two PopPK studies of fluoxetine were included in the final repository [25,26]. The detailed screening process is illustrated in Figure 1. The full text screen and the exclusion details are listed in Supplementary Table S1.

Study quality was evaluated using a standardized 33-item checklist (Supplementary Figure S1). The mean compliance score across the included studies was 90.91%. However, common reporting deficiencies were observed. No studies reported methods for handling missing data. Likewise, detailed covariate selection strategies, schematic diagrams of the final models, and comprehensive documentation of model-development steps were frequently omitted.

### 3.2. Literature Characteristics

All included studies were published from 2002 to 2011. For study populations, one study specifically enrolled lactating women with breastmilk sampling, enabling simultaneous modeling of plasma and milk concentrations [25]. Regarding dosing regimens, one study used weight-based dosing ([0.17–0.94] [25] mg/kg/day), whereas the pediatric study employed a flat dosing strategy (20 mg/day) [26,27]. Detailed study characteristics are summarized in Table 1.

All included PopPK models were developed using NONMEM and adopted the first-order conditional estimation method [25,26,27]. Although two studies detected the fluoxetine and norfluoxetine concentrations, no one study developed the parent–metabolite model. All models used a one-compartment model for the description of fluoxetine and appended an additional compartment to describe either breastmilk concentrations. The absorption rate constant (ka) varied markedly across the two studies, with 0.3 [25] and 0.666 [26,27] /h reported. By contrast, the apparent clearance (CL/F) was quite similar, whereas the apparent volume of distribution (V/F) showed substantial variability. Both models employed a proportional residual error model for unexplained variability.

Covariate assessment was constrained by the relative homogeneity of subject characteristics. In one model, body weight emerged as a significant covariate on V/F [26,27]. Both studies conducted an internal evaluation using GOF and VPC plots. One study performed an external evaluation using an independent dataset [25]. A summary of structural models, parameter estimates, variability components, and covariates is provided in Table 2.

### 3.3. Clinical Dataset Characteristics

The external evaluation cohort comprised 198 participants, most of whom were females (146, 73.7%). Participants were stratified into pediatric (<18 years; n = 102) and adult (≥18 years; n = 92) groups. The pediatric group had a median age of 15 years (range 12–17) and a median body weight of 55 kg (range 35.9–96), whereas the adult group had a median age of 21 years (range 18–56) and a median body weight of 59 kg (range 35.9–115). Overall, the cohort’s median age was 17 years (range 12–56), and the median body weight was 59 kg (range 35.9–115). The daily dosage in this cohort ranged from 20 to 40 mg. There were 241 fluoxetine plasma concentrations and 241 norfluoxetine plasma concentrations. The median observed concentration of fluoxetine was 140.97 ng/mL (range: 7.43–979.93 ng/mL), while the median observed concentration of norfluoxetine was 129.2 ng/mL (range: 9.6–490.41 ng/mL). The dataset included both adolescents and young adults, enabling the evaluation of model performance across developmental stages with distinct maturational and physiological profiles. Fluoxetine plasma concentrations were available for all participants, allowing direct comparison with model-predicted exposures.

### 3.4. External Evaluation

**Figure 2:**
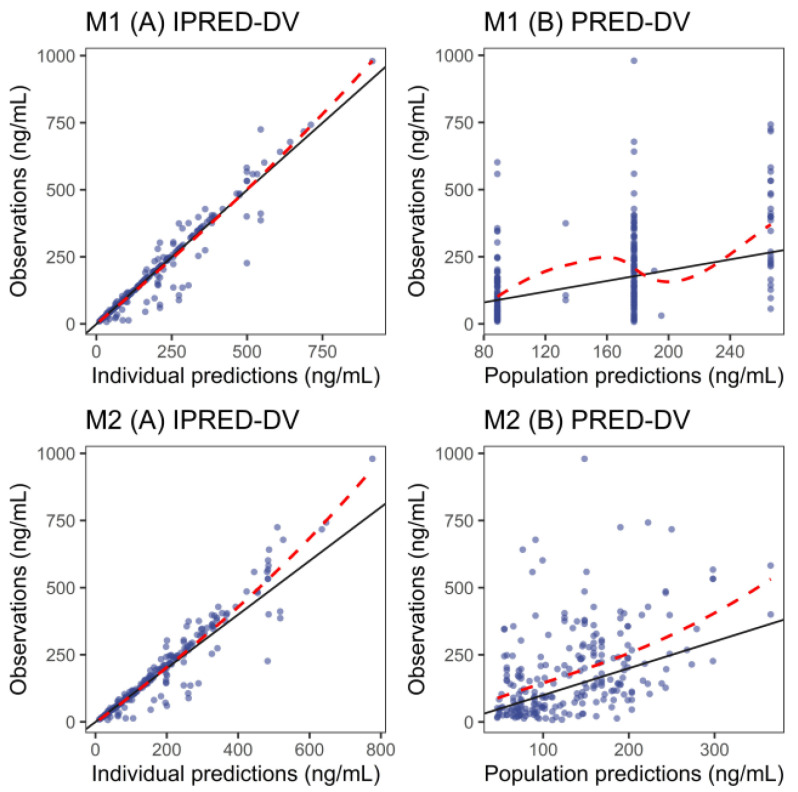
External validation of goodness-of-fit plots for all evaluated fluoxetine population pharmacokinetic models. Notes: M1 was the model developed by Panchaud et al. [25]; M2 was the model developed by Wilens et al. [26]. The red dashed lines represent the loess regression trendline and the black solid line means the reference line. The blue dots represent observed data.

**Figure 3:**
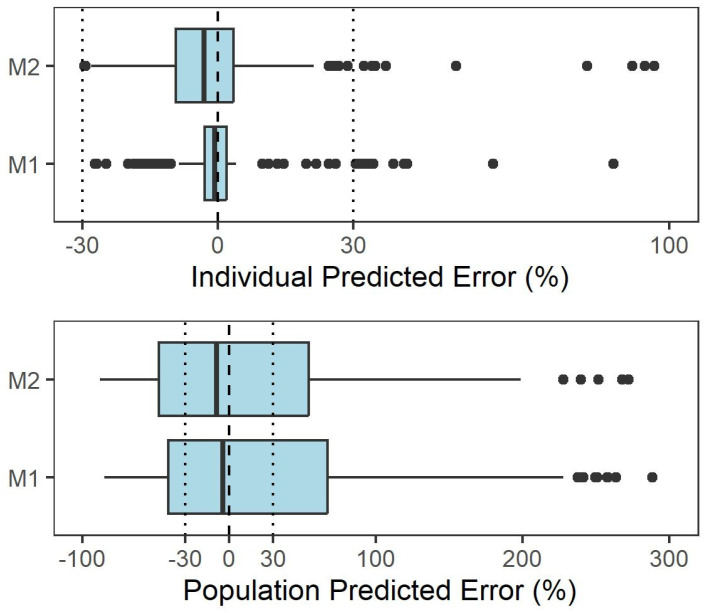
Prediction error distributions for individual-level (upper panel) and population-level (lower panel) predictions across the evaluated pharmacokinetic models. Notes: M1 was the model developed by Panchaud et al. [25]; M2 was the model developed by Wilens et al. [26,27].

The external evaluation GOF plots (Figure 2) showed that individual predictions aligned well with observed concentrations across the two models. However, population predictions tended to underestimate observed values. For individual and population predictions, median PEs were both within ±5% in the two models, which indicates that all models achieved acceptable accuracy in predictions. For MPE, the acceptable values were −2.66% and −0.43% for M1 and M2 under individual prediction and 50.7% and 78.32% under population prediction, respectively. A similar pattern was found for RMSE, with values of 8.43% and 2.57% for individual prediction and 189.87% and 248.65% for population prediction (Table 3). NPDE diagnostics (Supplementary Figure S2; Supplementary Table S1) were employed to assess predictive performance. Both models were shown to have prediction discrepancy using the Chinese dataset, while M2 showed better prediction performance compared with M1 (Figure 2 and Figure 3, and Supplementary Figure S2).

### 3.5. PopPK Model

**Figure 4:**
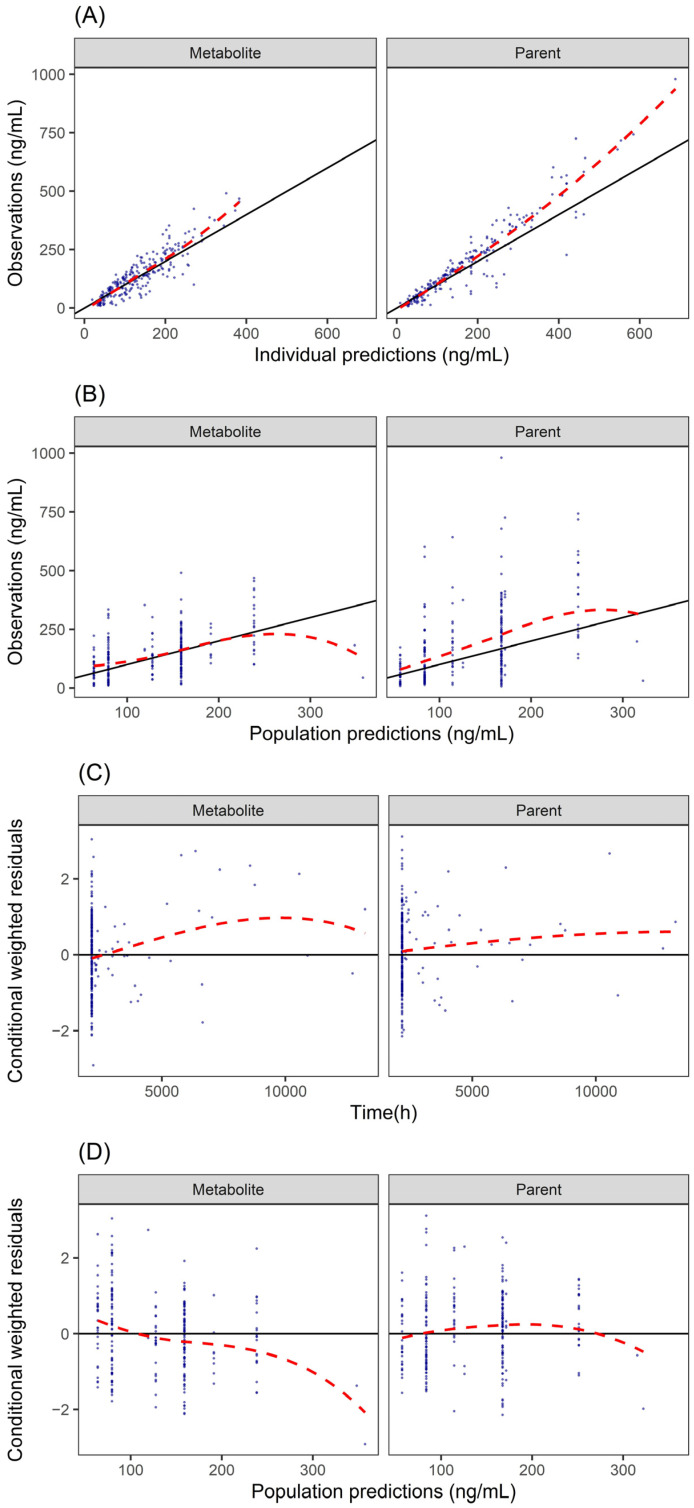
Goodness-of-fit plots for the final PopPK model. (**A**) the individual predictions vs the observation of the fluoxetine parent (right panel) and metabolite nor-fluoxetine (left panel); (**B**) the population predictions vs the observation of the fluoxetine parent (right panel) and metabolite nor-fluoxetine (left panel); (**C**) the conditional weighted residuals vs time plot of the fluoxetine parent (right panel) and metabolite nor-fluoxetine (left panel); (**D**) the conditional weighted residuals vs population predictions plot of the fluoxetine parent (right panel) and metabolite nor-fluoxetine (left panel). Notes: The solid line in the Observations versus PRED/IPRED plots represents the line of unity (y = x). The solid line in the CWRES versus PRED/TIME plots represents the horizontal line (y = 0). The dashed red line represents a smooth regression line. CWRES, conditional weighted residual; IPRED, individual-predicted concentration; PRED, population-predicted concentration.

The final joint parent–metabolite model for fluoxetine and norfluoxetine was parameterized as two connected one-compartment models, consistent with the structures in prior published PopPK models. The process of including and excluding covariates is shown in Supplementary Table S3. Model diagnostics supported an adequate fit. GOF plots (Figure 4) showed observations evenly distributed around the line of identity for both parent and metabolite without systematic bias. Prediction-corrected visual predictive checks (pcVPC; Supplementary Figure S3) captured the 5th, 50th, and 95th percentiles, and bootstrap medians with 95% CIs closely matched the final estimates (Table 4). For fluoxetine, CLP/F was 2.91 L/h, with a relative standard error (RSE) of 23%, and the central volume of distribution (VP/F) was 24.9 L (RSE: 38%). For norfluoxetine, CLM/F was 3.24 L/h (RSE: 20%), and VM/F was 1.52 L (RSE: 57%). IIV was moderate for CLP/F (31.6%) and CLM/F (20.9%), with low-to-moderate η-shrinkage. RUV for both the parent and metabolite was within acceptable limits. Sex emerged as the sole statistically significant covariate on fluoxetine clearance; clearance in males was estimated to be 16.5% higher than in females.

### 3.6. Optimized Dosage Regimens for Chinese Patients

**Figure 5:**
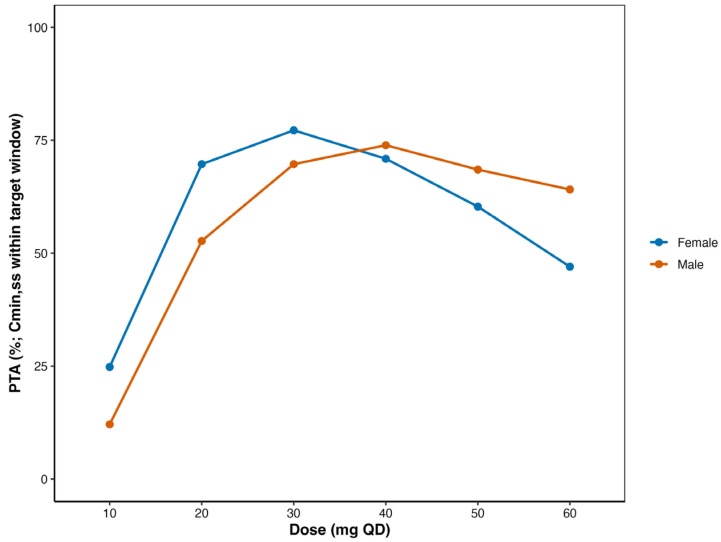
Probability of achieving target trough concentrations of active moiety.

Simulation results demonstrated that the PTA trough concentrations varied across analytes, dose levels, and sex (Figure 5). For the active moiety (total of fluoxetine and norfluoxetine), when administered to females at a dose of 20–40 mg/day, the PTA exceeded 70%, and when administered to males at a dose of 30–50 mg/day, the PTA also exceeded 70%. Median trough concentrations and IQR of fluoxetine, norfluoxetine, and the active moiety are summarized in Supplementary Table S4. Median trough levels increased approximately proportionally with dose for all three analytes. For the active moiety, median concentrations in females were higher than in males at equivalent daily doses, particularly at 60 mg/day (females: 502.32 ng/mL; males: 372.84 ng/mL). The simulation results are consistent with the actual Chinese clinical patient’s long-term drug administration concentration–density graph of the total active ingredient (Supplementary Figure S4). However, for patients receiving a 20 mg daily dose, the majority had an excessively low rate of achieving the target level. High-dose (50–60 mg QD) medication indicates that women are more likely to exceed the upper limit of the treatment range, which may lead to adverse reactions.

## 4. Discussion

This study represents a critical step toward precision dosing of fluoxetine in Chinese patients. We first conducted an external evaluation of previously published fluoxetine PopPK models using a real-world dataset from Chinese psychiatric patients, which revealed that the existing models did not adequately describe the pharmacokinetics in this population. Meanwhile, the existing models are unable to conduct an overall prediction of the parent drug and its metabolites. To address this, we developed a joint parent–metabolite model based on trough plasma concentrations from Chinese patients, demonstrating that males exhibited higher clearance than females. Using this population-specific model, we further performed dosage regimen simulations to provide optimized dosing recommendations for Chinese patients.

By establishing a model repository, this study provides an overview of the current stage of precision dosing research for fluoxetine. To date, all available population pharmacokinetic models have been developed in special populations, with one focused on perinatal women [25] and one in pediatric patients [26,27]. Fluoxetine was the first antidepressant approved for pediatric use. Although the U.S. FDA later introduced a black-box warning regarding the risk of suicidality in pediatric patients treated with SSRIs [28], fluoxetine remains the first-line option and is still widely prescribed in clinical practice. In our cohort, nearly 50% of patients were under 18 years of age, receiving a median steady-state dose of 40 mg/day (range: 20–60 mg/day). The most frequently prescribed regimens for Chinese pediatric patients were 20 mg/day and 40 mg/day, which are consistent with the FDA label recommendations [10]. Because fluoxetine is primarily metabolized by *CYP2D6* and *CYP2C19*, age-dependent variation in the maturation of these enzymes may influence drug disposition. Previous studies have shown that *CYP2C19* activity is detectable at birth but remains low during the neonatal period, rapidly increasing within the first few months of life and reaching near-adult levels by approximately 3–6 months of age [29]. In contrast, *CYP2D6* expression is minimal at birth but rises sharply within the first few weeks, achieving adult-equivalent activity between 3 months and 1 year of age [30]. Therefore, in individuals older than 6 years, both *CYP2C19* and *CYP2D6* are considered fully mature, and their metabolic capacities are comparable to those observed in adults. Taken together, these findings support the appropriateness of applying a pediatric PopPK model for external evaluation in our study population, where the majority of participants were adolescents with mature metabolic enzyme profiles.

For perinatal women, pharmacovigilance data from the FAERS database have highlighted significant safety signals related to pregnancy and neonatal outcomes [31]. Reported adverse events include fetal and neonatal exposures during pregnancy and lactation as well as health-related outcomes such as atrial and ventricular septal defects, preterm birth, and other drug-related fetal effects. These findings underscore the urgent need for precision dosing strategies and better characterization of fluoxetine exposure through the uterus–fetus and breastmilk transfer pathways. However, due to the lack of clinical data in this study, pregnant and postpartum women were not included in the current analysis.

For the covariates that influence fluoxetine exposure, a previous study identified weight as a significant covariate that influences fluoxetine exposure [26]. However, in this study, we did not detect weight as a significant covariate. This may be attributed to the fact that the dataset included in the model development shows a narrow weight distribution (IQR: 48.15–67.75). In this study, sex was identified as the only significant covariate; males exhibited a 16.5% higher fluoxetine CL/F than females. This difference may be attributed to higher metabolic enzyme activity in males. Previous studies have shown that men have greater *CYP2D6*-mediated metabolism than women; for example [32], clomipramine (a *CYP2D6* substrate) demonstrated higher clearance in men [33]. Although *CYP2C19* activity has generally been reported to show minimal sex-related differences, Laine et al. observed slightly higher metabolic activity in men (omeprazole metabolite-to-parent ratio: 0.52 in men vs. 0.48 in women) [34]. Similarly, Tamminga et al. found that men exhibited higher *CYP2C19* activity, as indicated by increased metabolism of (S)-mephenytoin [35]. However, due to data limitations in our study, further pharmacogenomic research is needed to confirm these findings. Other covariates detected in other PK studies were found as pharmacogenetics factors. The prediction discrepancy of the previously published model and the observed data in Chinese patients may contribute to many factors. Firstly, the population demographic characteristic was quite different. Wilens’ model [26] was developed based on a pediatric population, while our dataset included both adolescent and young adults; the exposure characteristics may be a discrepancy. Moreover, due to fluoxetine metabolism characteristics, it was significantly influenced by the *CYP2C19* and *CYP2D6* genotypes [36]. However, compared with Europeans, East Asian populations (including Chinese) carry higher frequencies of *CYP2C19* loss-of-function alleles (*2, *3) and distinctive *CYP2D6* activity profiles (e.g., high prevalence of *10 and *36, *10 tandem), factors that can push parent–metabolite ratios and total exposure away from Western expectations [12,14,37,38]. A controlled study in Chinese volunteers showed *CYP2C19* poor metabolizers had 46% higher fluoxetine C_max_ and 128% higher AUC vs. extensive metabolizers, directly illustrating genotype-dependent exposure shifts relevant to China [15]. Contemporary pharmacogenetic guidance from CPIC recommends genotype-informed selection or dose adjustment for SSRIs metabolized by *CYP2D6/CYP2C19*, with fluoxetine also acting as a clinically meaningful inhibitor of *CYP2D6* (and to a lesser extent *CYP2C19*), compounding genotype effects over time [11]. Together, these data explain why several Western-derived models underpredicted at the population level during our external checks and support the need for Chinese-tailored models and TDM.

Currently, PopPK studies of fluoxetine are relatively limited. In the present study, the estimated apparent clearance (CL/F) and apparent volume of distribution (V/F) were 2.91 L/h and 24.9 L, respectively, which differ markedly from those reported in two other studies [CL/F (L/h): 8.42 and 0.181*BW; V/F(L): 690 L and 37.4*BW] [25,26,27]. After body weight normalization, the weight-adjusted CL (0.053 L/h/kg) in our study remained lower than that reported in the other two studies (0.131 and 0.181 L/h/kg). Although previous reports have suggested that [39], after adjustment for body weight, pharmacokinetic differences between children and adults are unlikely to be clinically relevant. The apparent discrepancies in CL/F and V/F estimates across PopPK studies are more likely attributable to differences in study populations, sampling strategies, and model structures rather than true age-related differences in fluoxetine pharmacokinetics. Notably, the two prior models [25,26] were developed in more specific populations, including pregnant women, children, and adolescents. Moreover, only the parent drug models were established. In this study, we encompassed a broader and more heterogeneous population, which may partly explain the differences observed and can be better utilized for clinical diagnosis and treatment.

Real-world prescribing dosage for fluoxetine indicates broadly consistent dose ranges across populations, with adjustments based on age, comorbidity, and tolerability. In adults, treatment usually starts at 20 mg/day and may be titrated to 30–40 mg/day [40], while higher doses are rarely used. In adolescents and children, doses of 10–40 mg/day are typical with weight-based adjustment, and lower starting doses are often preferred in elderly or hepatically impaired patients [8]. When exposure–response is uncertain, TDM and *CYP2D6* genotype information can help guide individualized dosing. For adult Chinese patients, our simulations indicate that 30–40 mg QD often maximizes the PTA of the recommended steady-state active-moiety trough range, with males more frequently requiring the higher dose. The dosage of 20 mg QD is likely subtherapeutic for a substantial proportion, whereas 60 mg QD increases the risk of exceeding the upper limit. Furthermore, the 30–40 mg dosage not only emerged as the optimal range in our simulations but also aligns with the commonly prescribed maintenance doses observed in real-world clinical practice, suggesting good feasibility and practical applicability clinically. This concordance provides external validation for the model predictions and supports 30–40 mg as a more rational recommended dose range for Chinese adult patients.

There are several limitations to this study. First, the relatively homogeneous study population may have restricted the identification of important covariates, such as age and body weight, and the lack of certain covariates, such as liver function indices, may have limited the clinical applicability of the model to some extent. Second, due to the retrospective study design, all samples were collected at trough concentrations, which may limit the robustness of model development, particularly with respect to accurately characterizing the absorption rate. Fixing ka (0.3 h^−1^) to a literature value was necessary given trough-only sampling [25]. These results align with reports that structural joint models and relatively sparse covariate effects often suffice for steady-state TDM applications of SSRIs [21]. Prospective, model-informed TDM cohorts in Chinese sites should test dose-exposure-response links and validate concentration thresholds locally. Ensemble model-averaging strategies across our repository may reduce model-specific bias in clinical tools [21,23]. Future prospective clinical studies with independent pharmacokinetic data are warranted to externally validate the model and further assess its applicability in clinical dosing optimization. Embedding these models in a bedside app with clear interpretation could close the loop between modeling and routine care.

## 5. Conclusions

The model reported in the literature performed poorly in external evaluation for Chinese patients. A joint parent–metabolite PopPK model with a sex effect on fluoxetine clearance explained steady-state troughs. Monte Carlo simulation results showed that 20–40 mg QD for females and 30–50 mg QD for males could maximized active-moiety PTA for adults. Implementation of MIPD has the potential to increase target attainment, improve clinical response, and reduce avoidable adverse effects in routine fluoxetine therapy.

## Figures and Tables

**Table 1:** Characteristics of published fluoxetine population pharmacokinetic studies and the external evaluation dataset.

Study (Publication Year)	Country (Type of Study)	Number of Subjects (M/F)	Number of Observations	Sampling Schedule (h)	Age Mean ± SD Median [Range]	Weight (Kg) Mean ± SD Median [Range]	Dose [Range]	Bioassay [LOQ]
Panchaud (2011) [25]	Study1: Australia Study2: Canada	Study1: Women: 14 Infants: 14 (7/7) Study2: Women: 10 Infants: 11	Maternal plasma: 49 Breastmilk: 112	Study1: Four women: 1, 2, 3, 4, 6, 8, 12, 24 h; ten women: one sample (1.1–23.5 h) to collect milk and plasma Study2: 2, 5, 8, 12, and 24 h to collect milk sample	Maternal: 31.8 [22.7–44.0] years Infant: 6.3 [0.12–25] months	Maternal: 64.5 [31–85] Infant: 5.3 [2.8–10]	Study1: 0.51 [0.24–0.94] mg/kg/day Study2: 0.39 [0.17–0.85] mg/kg/day	Study1: HPLC, 15 ug/L Study2: Gas-liquid chromatography, 1 ng/mL
Wilens (2002) [26,27]	America	Pediatrics: 21 (11/10)	Plasma: 168	6–10 samples per subject, 8–12 h post-dose	12.6 [6.0–17.0] years	Children: 39.9 ± 12.8 Adolescents: 67.3 ± 16.1	20 mg QD	LC–MS/MS 1.0 ng/mL
External dataset	China	198 (Women: 146; Men: 52)	241 fluoxetine and 241 norfluoxetine plasma concentrations	All concentration was trough concentration	17 [12–56]	59 [35.9–115]	20–60 mg QD	HPLC–MS/MS [1 ng/mL]

Notes: This table summarizes the general characteristics of the included studies, including publication year, study country, study population, biological matrices (plasma and/or milk), and analytical method. Abbreviations: LOQ, lower limit of quantification; HPLC–MS/MS, high-performance liquid chromatography–tandem mass spectrometry.

**Table 2:** Population pharmacokinetic structure and parameters of included studies.

Study (Publication Year)	Software (Algorithm)	Model	Fixed Effect Parameters		Between-Subject Variability (%CV)	Residual Unexplained Variability Prop% Add (mg/L)	External Validation	Model Application
Panchaud (2011) [25]	NONMEM VI (FOCE-I)	One-compartment model with first-order absorption, fixed ka = 0.3, MPR (milk-to-plasma ratio) applied as a scaling factor for milk compartment	CL/F (L/h)	=8.42	38.00%	Prop.err Plasma: 8% Milk: 37%	59 individuals from 8 external studies (MPR validation)	To predict infant drug exposure through breastfeeding
			V/F (L)	=690	/			
			Ka (/h)	=0.3 FIX	/			
			MPR	=0.59	32.00%			
Wilens (2002) [26,27]	NONMEM V (FOCE)	One-compartment model with first-order absorption and elimination	CL/F (L/h)	=0.181 × BW	52.00%	Prop.err: 18%	No	To characterize fluoxetine pharmacokinetics in pediatric patients (children and adolescents) with depression or OCD
			V/F (L)	=37.4 × BW	20.50%			
			Ka (/h)	=0.666 FIX	/			

Note: This table describes the pharmacokinetic structural model for fluoxetine and its active metabolite norfluoxetine, estimation methods, covariates, random effects components (inter-individual variability [IIV], residual unexplained variability [RUV]), and model evaluation techniques. Abbreviations: CL, clearance; V, volume of distribution; Ka, absorption rate constant; FOCE-I, first-order conditional estimation with interaction.

**Table 3:** Prediction error of the individual prediction and population prediction to observations for the evaluated models.

	IPRED			PRED		
Model	Median PE (%)	MPE (%)	RMSE (%)	Median PE (%)	MPE (%)	RMSE (%)
M1	−2.99	−2.66	8.43	−2.48	50.7	189.87
M2	−0.52	−0.43	2.57	2.72	78.32	248.65

Abbreviation: PE, prediction error; MPE, mean relative error; RMSE, root mean square error; IPRED, individual prediction; PRED, population prediction. Notes: M1 was the model developed by Panchaud et al. [25]; M2 was the model developed by Wilens et al. [26,27].

**Table 4:** The PK parameters with bootstrap results for the final popPK model.

Compounds	Parameter	Estimate (RSE%) [Shrinkage]	Bootstrap Median [95% CI]
Fluoxetine	CL/F, L/h	2.91 (23%)	2.76 [1.53–4.13]
	Sex effects on CL/F ^1^, %	16.50 (44%)	16.43 [6.64–27.21]
	V/F, L	24.9 (38%)	22.76 [9.06–38.48]
	Ka (fixed) h^−1^	0.3	/
	FM (fixed) ^1^	1	/
	IIV CL/F, %	31.6 (27%) [10%]	30.67 [23.39–37.51]
	Prop.err.sd, %	34.1 (12%) [20.8%]	34.64 [27.86–40.35]
	Add.err.sd, ng/mL	14.9 (49%) [20.8%]	14.84 [8.74–21.48]
Norfluoxetine	CL/F, L/h	3.24 (20%)	3.06 [1.77–4.53]
	V/F, L	1.52 (57%)	1.17 [0.67–1.98]
	IIV CL/F, %	20.9 (41%) [48%]	21.01 [4.96–29.46]
	Prop.err.sd, %	30.5 (16%) [37.3%]	29.38 [19.87–40.01]
	Add.err.sd ng/mL	22.9 (31%) [37.3%]	23.20 [13.28–34.44]

Abbreviations: inter-individual variability (IIV); apparent clearance (CL/F); apparent volume of distribution (V/F); absorption rate constant (Ka); sex effects on CL, the females influence on the clearance; the fraction of metabolism from fluoxetine to norfluoxetine (FM); Prop.err.sd, the proportional error; Add.err.sd, the additive error; RSE: relative standard error. ^1^ CL/F_,males_ = CL/F,_females_ × (1 + 0.165)^Males^; Females = 0; Males = 1.

## Data Availability

The original contributions presented in this study are included in the article/Supplementary Material. Further inquiries can be directed to the corresponding authors.

## Supplementary Materials

The following supporting information can be downloaded at: https://www.mdpi.com/article/10.3390/pharmaceutics17121516/s1, Figure S1: Risk-of-bias assessments of fluoxetine PopPK studies. Figure S2: The normal prediction distribution errors plots. Figure S3: The prediction corrected visual predictive check plot of the final popPK model. Figure S4: Distribution dentisy of therapeutic drug concentrations in the Chinese population. Table S1: The 15 full text screen and the exclusion details. Table S2: The normalized prediction distribution errors statistic test. Table S3: Covariate forward inclusion and backward exclusion. Table S4: PTA and steady-state trough concentrations by analyte, sex.
