# Supplementary material for: Bridging Literature and Real-World Evidence: External Evaluation and Development of Fluoxetine Population Pharmacokinetics Model
Source: Pharmaceutics. 2025 Nov 24;17(12):1516. doi: 10.3390/pharmaceutics17121516 (PMC12736537; doi:10.3390/pharmaceutics17121516)
Supplement: Supplementary file 1 [file pharmaceutics-17-01516-s001.zip › pharmaceutics-3878329-supplementary.pdf]

Bridging literature and real-world evidence: external evaluation and optimization of fluoxetine population pharmacokinetics model

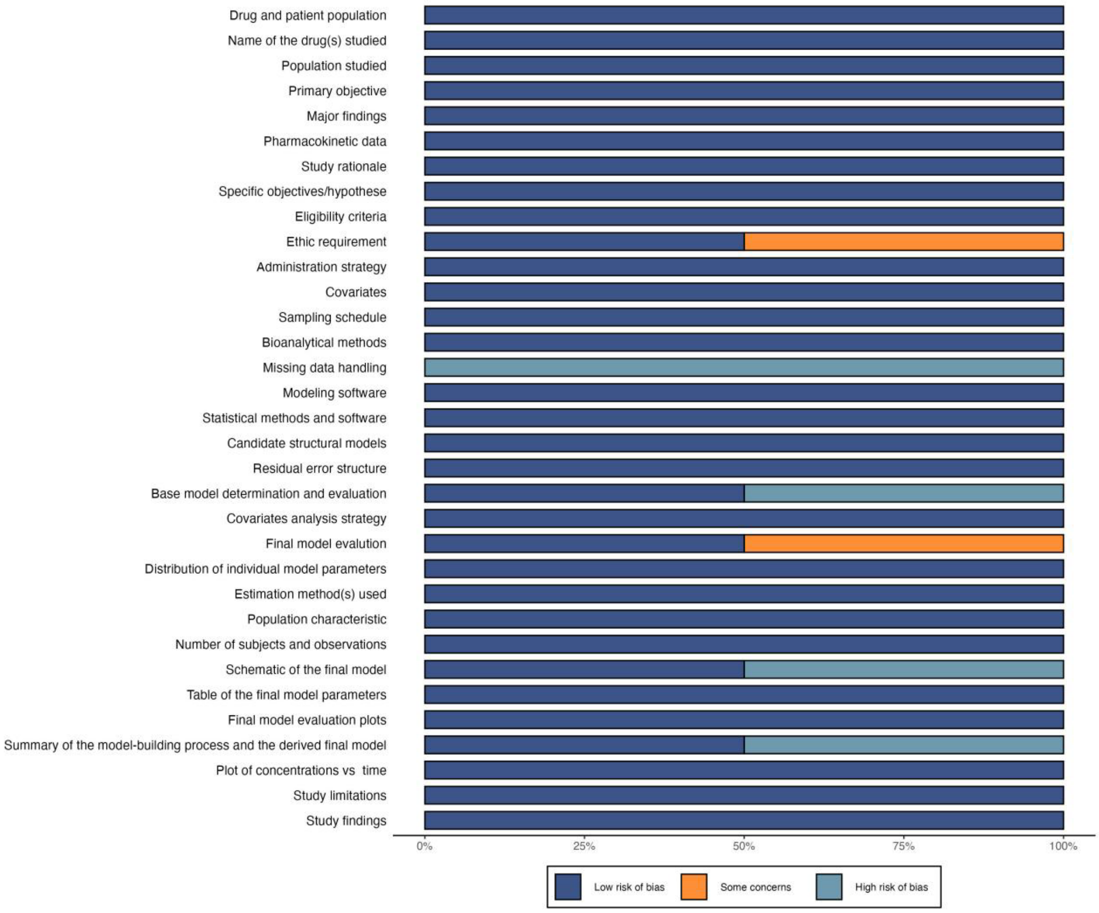

Figure S1. Risk-of-bias assessments of fluoxetine PopPK studies.

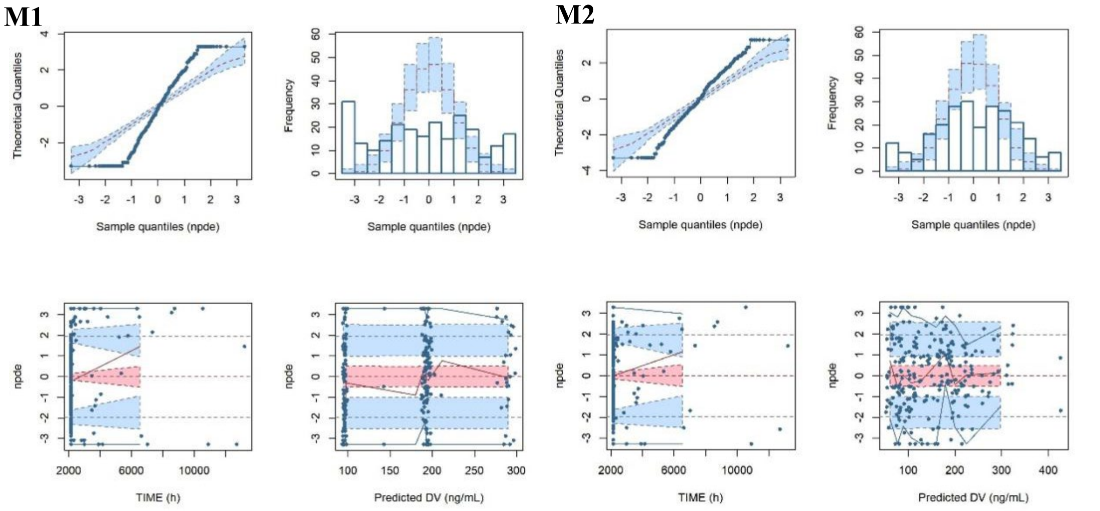

Figure S2. The normal prediction distribution errors plots.

Notes: M1 developed by Panchaud 2011; M2 developed by Wilens 2002.

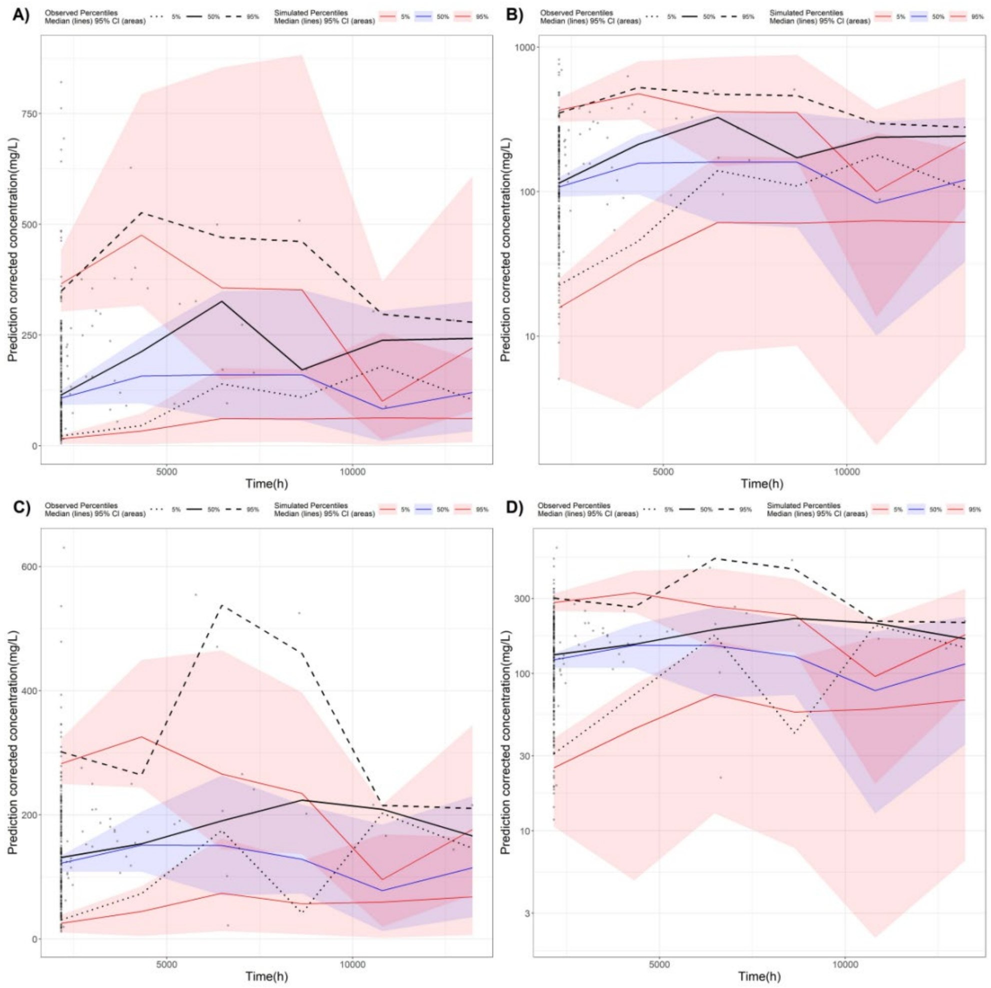

Figure S3. The prediction corrected visual predictive check plot of the final popPK model.

A) parent continuous scale; B) parent semi-log scale; C) metabolite continuous scale; D) metabolite semi-log scale.

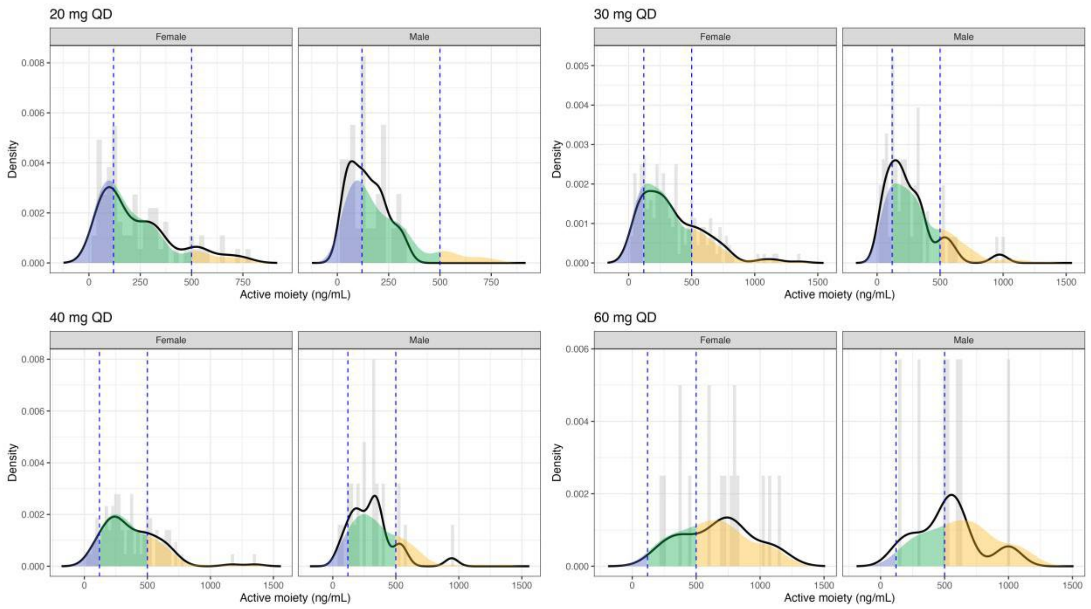

Figure S4. Distribution dentisy of therapeutic drug concentrations in the Chinese population.

Note: Purple: Below the therapeutic range; Green: Within the therapeutic range; Yellow: Outside the therapeutic range; Blue dotted line: Treatment range [120 - 500] ng/mL; Black solid line: Distribution density curve

Table S1. The 15 full text screen and the exclusion details

Author	Publication year	Excluded Reason	Comments
Cun Zhang	2024	No PopPK Model	Although this study analyzed the DDI interaction of aripiprazole and fluoxetine when used in combination based on NONMEM analysis, the main drug in the study was aripiprazole, and fluoxetine was only examined as a covariate (whether the drugs were used in combination) without describing the PopPK model of fluoxetine, and thus was excluded.
Julia Izsak	2024	No PopPK Model	This study is a pure PK analysis. It mainly examined the impact of different obesity conditions on drug PK through stratified analysis. Only statistical analysis was conducted, and no medication recommendations were given. This is not a report of the PopPK model.
Amelia Nathania Dong	2020	No PopPK Model	This article is a study on enzyme kinetics. The main focus was to examine the effects of different genotypes of CYP450 2C19 on the probe (fluoxetine). Although pharmacokinetic changes were investigated, model analysis was not included.
Samantha Luk	2014	No PopPK Model	This study mainly investigated the effects of CYP2C19 and fluoxetine inhibition on the renal distribution of diazepam. Although keywords such as fluoxetine and PopPK were included, no fluoxetine PopPK model was reported and was thus excluded.
Reo Tanoshima	2013	Sample: Breast	Although this study established a PopPK model for fluoxetine based on NONMEM, since the PK samples were derived from breast milk, no breast milk samples were collected in this study, making it impossible to conduct external validation for this model. Therefore, it was excluded.
Reo Tanoshima	2013	Sample: Breast	This study is a methodological introduction to the construction of the fluoxetine milk PopPK model by the same author. The content is basically the same. It does not include plasma samples and cannot be verified, so it has been excluded.

H. C. Wang	2011	No PopPK Model	This study mainly investigated the effects of CYP2C19 and fluoxetine inhibition on the renal distribution of diazepam. Although keywords such as fluoxetine and PopPK were included, no fluoxetine PopPK model was reported and was thus excluded.
M. Kandasamy	2010	No PopPK Model	This study is a retrospective analysis of the fluoxetine bioequivalence study in the Indian population. It only conducted a non-atrial analysis on the population and did not establish a PopPK model. Therefore, it was excluded.
B. Mannheimer	2009	No PopPK Model	This study was aimed at evaluating the exposure-effect relationship of fluoxetine, mainly assessing the safety risks for patients after using fluoxetine and other treatments. No PopPK model was established.
V. P. Sinha	2006	Missing Key PK parameters	The study is a conference abstract. Although a plasma population PK model for the parent drug and its metabolites was established based on NONMEM, only typical values of the main model parameters were provided, and no other information was given. Therefore, it is impossible to reconstruct the model based on the available data.
D. N. Juurlink	2005	No PopPK Model	This study mainly analyzed the clinical toxicity practices of combined use of digoxin and fluoxetine, etc. However, no PopPK model was constructed for fluoxetine in this study.
P. J. Grimm	2001	No PopPK Model	This study only focused on the clinical safety of fluoxetine use, and did not construct a PopPK model based on the PK behavior of fluoxetine.
D. Clark	2000	No PopPK Model	This study focus on the side effects of the fluoxetine in different genotypes, and did not develop the population pharmacokinetic model of fluoxetine.

Table S2. The normalized prediction distribution errors statistic test.

Model	Wilcoxon signed-rank test(p)	Fisher variance test(p)	Shapiro-Wilk test(p)	Global adjusted(p)
M1	0.195	0 ***	7.14e-07 ***	0 ***
M2	0.508	0 ***	0.00779 ***	0 ***

Notes: p value are indicated as *represents p<0.05; ** represents p< 0.01; ***represents p< 0.001.

M1 developed by Panchaud 2011; M2 developed by Wilens 2002.

Table S3. Covariate forward inclusion and backward exclusion

Model	Base OFV	Drop OFV	GOAL	Significant P
Forward inclusion	First cycle			
CL_Age	4702.64254	1.80034	6.63490	0.179670
CL_SEX	4702.64254	11.56710	6.63490	0.000671
CL_WT	4702.64254	Failed	6.63490	/
CLM_AGE	4702.64254	0.37803	6.63490	0.538660
CLM_SEX	4702.64254	4.00359	6.63490	0.045403
CLM_WT	4702.64254	Failed	6.63490	/
Forward inclusion	Second cycle			
CL_Age	4691.07544	3.18681	6.63490	0.1074235
CL_WT	4691.07544	Failed	6.63490	/
CLM_AGE	4691.07544	0.18665	6.63490	0.665720
CLM_SEX	4691.07544	1.38566	6.63490	0.239140
CLM_WT	4691.07544	Failed	6.63490	/
Backward exclusion				
CL_SEX	4691.07544	-11.39552	10.82800	0.000736

Abbreviation: CL: the parent compound clearance; CL_M: the metabolites compound clearance; OFV: Objective function value.

Table S4. PTA and steady-state trough concentrations by analyte, sex.

SEX	Dose	N	Target	PTA	Median	Q1	Q3
Female	10	1000	Active moiety	24.8	83.72	54.61	119.72
Female	20	1000	Active moiety	69.7	167.44	109.23	239.43
Female	30	1000	Active moiety	77.2	251.16	163.84	359.15
Female	40	1000	Active moiety	70.9	334.88	218.45	478.87
Female	50	1000	Active moiety	60.3	418.60	273.06	598.58
Female	60	1000	Active moiety	47	502.32	327.68	718.30
Male	10	1000	Active moiety	12.1	62.14	38.79	92.17
Male	20	1000	Active moiety	52.7	124.28	77.57	184.35
Male	30	1000	Active moiety	69.7	186.42	116.36	276.52
Male	40	1000	Active moiety	73.9	248.56	155.15	368.69
Male	50	1000	Active moiety	68.5	310.70	193.94	460.86
Male	60	1000	Active moiety	64.1	372.84	232.72	553.04
Female	10	1000	Fluoxetine	11.9	42.99	26.36	68.11
Female	20	1000	Fluoxetine	44.3	85.97	52.72	136.22
Female	30	1000	Fluoxetine	58.3	128.96	79.09	204.33
Female	40	1000	Fluoxetine	60.2	171.95	105.45	272.43
Female	50	1000	Fluoxetine	54.6	214.94	131.81	340.54
Female	60	1000	Fluoxetine	49.4	257.92	158.17	408.65
Male	10	1000	Fluoxetine	5.7	29.40	17.14	48.83
Male	20	1000	Fluoxetine	27.2	58.81	34.27	97.66
Male	30	1000	Fluoxetine	45.2	88.21	51.41	146.49
Male	40	1000	Fluoxetine	53.1	117.61	68.54	195.31
Male	50	1000	Fluoxetine	57.7	147.02	85.68	244.14
Male	60	1000	Fluoxetine	55.2	176.42	102.82	292.97
Female	10	1000	Norfluoxetine	5.7	39.45	27.98	53.29
Female	20	1000	Norfluoxetine	58	78.90	55.97	106.58
Female	30	1000	Norfluoxetine	81.6	118.35	83.95	159.87
Female	40	1000	Norfluoxetine	82.1	157.80	111.93	213.16
Female	50	1000	Norfluoxetine	69.8	197.25	139.91	266.45
Female	60	1000	Norfluoxetine	55.5	236.70	167.90	319.74
Male	10	1000	Norfluoxetine	2.3	31.85	21.45	44.57
Male	20	1000	Norfluoxetine	39.9	63.70	42.89	89.15
Male	30	1000	Norfluoxetine	67.5	95.55	64.34	133.72
Male	40	1000	Norfluoxetine	78.5	127.41	85.79	178.29
Male	50	1000	Norfluoxetine	74.4	159.26	107.24	222.87
Male	60	1000	Norfluoxetine	67.1	191.11	128.68	267.44

Note: N: the number of simulation; PTA: probability of target attainment; Q1: first trough concentration quartile; Q3: third trough concentration quartile; Active moiety, was the sum of the

concentration of fluoxetine and the norfluoxetine.
